# Improving the management of people with a family history of breast cancer in primary care: before and after study of audit-based education

**DOI:** 10.1186/1471-2296-14-105

**Published:** 2013-07-24

**Authors:** Imran Rafi, Susmita Chowdhury, Tom Chan, Ibrahim Jubber, Mohammad Tahir, Simon de Lusignan

**Affiliations:** 1 Division of Population Health Sciences and Education, St George’s, University of London, Cranmer Terrace, London SW17 0RE, UK; 2 Clinical Innovation and Research Centre (CIRC), Royal College of General Practitioners, 30 Euston Square, London NW1 2FB, UK; 3 PHG Foundation, 2 Worts Causeway, Cambridge, Cambridgeshire CB1 8RN, UK; 4 Department of Healthcare Management and Policy, University of Surrey, Guildford, Surrey GU2 7XH, UK

## Abstract

**Background:**

In England, guidance from National Institute for Clinical Excellence (NICE) states women with a family history of breast cancer presenting to primary care should be reassured or referred.

We reviewed the evidence for interventions that might be applied in primary care and conducted an audit of whether low risk women are correctly advised and flagged.

**Methods:**

We conducted a literature review to identify modifiable risk factors. We extracted routinely collected data from the computerised medical record systems of 6 general practices (population approximately 30,000); of the variables identified in the guidance. We implemented a quality improvement (QI) intervention called audit-based education (ABE) comparing participant practices with guidelines and each other before and after; we report odds ratios (OR) of any change in data recording.

**Results:**

The review revealed evidence for advising on: diet, weight control, physical exercise, and alcohol. The proportion of patients with recordings of family history of: disease, neoplasms, and breast cancer were: 39.3%, 5.1% and 1.3% respectively. There was no significant change in the recording of family history of disease or cancer; OR 1.02 (95% CI 0.98-1.06); and 1.08 (95% CI 0.99-1.17) respectively. Recording of alcohol consumption and smoking both increased significantly; OR 1.36 (95% CI 1.30-1.43); and 1.42 (95% CI 1.27-1.60) respectively. Recording lifestyle advice fell; OR 0.84 (95% CI 0.81-0.88).

**Conclusions:**

The study informs about current data recording and willingness to engage in ABE. Recording of risk factors improved after the intervention. Further QI is needed to achieve adherence to current guidance.

## Background

Breast cancer is the most common cancer in women and accounts for over 30% of new cancers in females, in the UK (Additional file 1: Box S1) [1-3]. National Institute for Health and Clinical Excellence (NICE) guidelines for familial breast cancer highlight the importance of recording family history as a predictor of risk for breast cancer. The key elements of the NICE guidelines are that people with a family history should be given lifestyle advice about their breast cancer risk including information about: hormone replacement therapy (HRT) and oral contraceptives, lifestyle (including diet, alcohol, etc.), breastfeeding, family size and timing. The guidance also suggests that people with modifiable risk factors should have these managed in primary care. The level of risk depends on: the number of affected relatives, closeness of relationships and the age at which the relatives developed breast cancer; the guidelines recommend the use of information from family history which should be taken into account in assessing risk and in deciding whether and when to refer, and when to reassure [4].

Primary care has a role in prevention, promoting increased awareness, detecting presentation early as well as referral of suspected cancers. Reduction in risk through prevention is a core function of primary care. Additionally, early detection and management of cancers is important and clearly, recording of family history may help clinicians consider a diagnosis of cancer, stratify risk and make an appropriate decision about referral. The UK Cancer Reform Strategy [5] supports early diagnosis. The Royal College of General Practitioners (RCGP) National Audit of Cancer Diagnosis reported that 87% of cases presented with symptoms within primary care [6].

Many women consult their general practitioners (GP) each year with concern over breast cancer risk. 40–50 people with a positive family history of breast, ovarian or bowel cancer are found on a GPs list of 2000 patients [7]. Women with increased risk based on a positive family history as described within NICE guidelines may be advised to undergo a range of different forms of surveillance, genetic testing or even preventive management [8]. However, many can be reassured that their risk is not increased above that of the general population. These consultations also provide an opportunity to record non-modifiable risk of the woman and to give risk-reducing advice such as modification of lifestyle and environmental factors.

Audit, seeks to improve patient care and outcomes through a systematic review of care against explicit criteria and is an established method of promoting quality improvement in the care of patients. The Royal College of Physicians report “Teams without Walls” calls for greater use of audit as a means of continuous improvement [9]. English primary care lends itself to conducting clinical audit [10]. There is a registration based system where individuals can only register with one practice, and that practice acts as a gatekeeper to secondary care. Everyone has a national unique identifier, an NHS number, which helps identify their records. Primary care is universally computerised, nearly all clinicians complete computer records at the time of the consultation. Many practices have computerised since the 1990s; with data quality improving considerably after pay-for-performance based on computerised records was introduced in 2004 [11,12].

We carried out this study to determine whether an audit-based education improved risk factor management in women who have a low risk of breast cancer; using improved recording of family history and its risk factors in primary care computerised medical record systems as our audit criteria. The focus on the audit was to record family history and record risk factors where there was evidence that intervention in primary care affects outcome.

## Methods

### Overview

This is a before and after study of audit-based education (ABE), a non-judgemental quality improvement intervention to explore concordance with guidance in the management of people with a family history of breast cancer in primary care. The study consisted of two phases: (1) A literature review to identify the lifestyle factors that may reduce the risk of breast cancer, which might form part of a quality improvement (QI) initiative in this domain. The objective of the literature review was to inform what elements of the NICE guidance might be amenable to interventions carried out in primary care; (2) Piloting the audit-based education process developed in the first phase in six volunteer practices.

### Literature review to explore the association of lifestyle factors with breast cancer

The literature review was conducted to identify the risk factors associated with breast cancer. A comprehensive search of numerous databases was carried out. The searches were limited to human subjects, English language and studies from the last ten years. The searches identified 1119 articles including 312 reviews. The initial search identified articles prior to 1/1/2010, and was used to inform the components of the audit based education programme. It has subsequently been reviewed to include major literature published since the start of the study.

Inclusion criteria;

•Data representative of the general population.

•Articles which discuss modifiable risk factors at population level risk.

•Articles only related to breast cancer and not breast cancer-associated problems (e.g. lymphedema in breast cancer patients).

•Studies of risk assessments with confidence intervals or at least P values.

Exclusion criteria:

•Small sample size (n) less than 50.

•Articles in relation to other issues such as familial risk factors and diagnostic tests (e.g. mammography).

The outputs of the searches were sorted based on title and abstract into those to be definitely excluded and those to retain. Findings from about 66 full texts which included systematic reviews and primary articles were used for the literature review. (A full copy of the literature review is available online at: http://www.clininf.eu/fhbc).

### Audit process

We developed an audit based education (ABE) intervention to support this process (Additional file 1: Box S2) [13]. ABE is a quality improvement (QI) intervention developed over the last 15 years by SdeL et al., which provides general practice based education, peer support and documents the gap between achievement and guidelines. In observational studies this intervention improved the quality of cardiovascular disease management [14,15], and has also been used in a quality improvement trial [16]; where it has produced a modest but statistically significant drop in systolic BP compared with usual practice [17]. The theoretical basis for this intervention is that audit, feedback and professional meetings are known to have small but positive effects on the quality of data recording [18,19]. Any change in data coding that takes place might be explained by *control theory,* which suggests that improvements in the quality of data recording is most likely if feedback is accompanied by a target or action plan, ideally in writing [20].

In this study, the educational component included briefing about the importance of breast cancer and its family history (Additional file 1: Box S1 gives an overview). Additionally, we designed data extraction queries based on the output from the literature review. As family history and risk factors can be represented by a wide range of codes we used an established method to ensure we comprehensively extracted the relevant data. The variable list extracted is available on-line at: http://www.clininf.eu/projects/fhbc.

We extracted data from general practice EPR systems using Morbidity Information Query and Export SynTax (MIQUEST), a data extraction method sponsored by the Department of Health of England. We processed these data using SPSS (Version 18) for statistical analysis [21]. We report the completeness of recording of audit variables, demographics and key co-morbidities. We planned to eventually develop self-audit tools for general practice similar to those we had developed to identify errors in the coding of diabetes [22,23]. Any inconsistencies between in coding between baseline and subsequent collection; were corrected by creating a mapping between sets (Additional file 1: Table S2).

### Pilot audit based-education

We collected initial data from six volunteer practices in South London and fed back these data to representatives of the pilot practices. The practices developed an action plan. This involved them deciding how they might raise general awareness of family history recording, and recording of lifestyle factors by feeding back the presentations from the baseline meeting, as well as discussing the workshop outputs at meetings held at general practices. The key points from the workshop were noted down and lead GPs who agreed to disseminate through their usual practice business processes. We then conducted a second data collection process with these practices, and then fed this data back to complete the audit cycle.

The data are reported as two cross-sections at two time periods, approximately 6 months apart. We used two cross sections at two different time points so that the data included recording of lifestyle factors about new patients who registered during the audit period. We hypothesised that a practice responding to the intervention might be most likely to ask new patients about any family history of breast disease.

We used Altman’s method to assess any differences between before and after readings; this method is designed to be used to compare two cross sections measured at different time points [24]. We have reported crude and standardised rates of recording. The participant general practitioners agreed an action plan at the baseline presentation, which was shared with the rest of their practice. We report the characteristics, such as patient demographics, of the practices using descriptive statistics: mean and standard deviation (SD). We compared comparable data sets using Pearson Chi-square to report differences in proportion, and we used odds ratios (OR) and 95% confidence intervals to explore whether data recording by general practitioners had increased from pre-to post audit.

A baseline data collection (round 1) was made to investigate the extent to which NICE guidance was being followed in terms of the provision of lifestyle advice to women with a low risk of breast cancer who can be reassured and managed in general practice. We planned a data quality workshop (DQW) which is probably the most important component of the QI process; it is an interactive process involving representatives of each practice. The objective of the DQW was twofold: (1) To share baseline levels of recording between general practices and their general practitioners; (2) To recognise the value of recording family history and implementing an action plan for improved recording and management. The second data collection (round 2) served to measure any change in the quality of data recording after six months. An Example of a DQW presentation is available online at: http://www.clininf.eu/fhbc.

### Ethical considerations

This intervention meets the National Patient Safety Agency (NPSA) and National Research Ethics Service (NRES) descriptions of clinical audit [25]. Hence ethics approval was not sought. There was no attempt to influence the decisions of clinicians and patients.

## Results

### Overview

Although there are many risk factors associated with a family history of breast cancer; there is a much more limited number where intervention is known to affect outcome, which we included in the audit. Whilst recording of the numbers of cases of a family history of breast cancer did not increase, the odds ratio of recording risk factors where interventions might affect outcome did. However, practitioners felt they lacked knowledge of how best to record. Also they felt that their computer system did not readily support recording of family history.

### Findings from literature review about risk factors associated with breast cancer

The broad consensus from the literature review about risk factors associated with breast cancer is summarised below. These formed the “long-list” of potential variables that might be included in our audit–based education, quality improvement intervention.

1. **Oral contraceptive pills (OCP).** There is evidence of an increase in the risk of breast cancer during use of OCP with higher dosage being related to higher risk [26-29]. After cessation of treatment the risk gradually decreases over time and disappears about 10 years after discontinuation of OCP [26,27].

2. **Hormone replacement therapy (HRT).** Long term HRT apparently increases breast cancer risk especially when used from 50 years of age [30]. The risk is greatest for combined oestrogen-progestogen preparations (CHRT)[26,31,32] and lowest for oestrogen-only preparations of HRT (ERT) [33,34]. This effect is limited when Body Mass Index (BMI)^5^ is less than 25 kg/m^2^. According to the recommendation by the British Menopause Society, HRT may be given for symptom relief in the short term (up to 5 years) but long term use needs to be assessed individually at regular intervals [35].

3. **Breastfeeding.** Breastfeeding is reported to be protective for breast cancer [36,37]. Decreased risk is related to average length of time of breastfeeding [38,39]. Risk of breast cancer seems to decrease by approximately 4.3% for every 12 months of breastfeeding [27].

4. **Alcohol.** It is reported that there is about a 10% increased risk of breast cancer for each additional unit (10–12 g) of alcohol per day [40]. Risk appears to be 28-50% greater for those who typically consume approximately two drinks per day compared to those who do not drink [41-44]. Consumption of one drink per day or less does not significantly affect risk of breast cancer [45-47]. There is no difference in risk based on type of alcohol consumed [48].

5. **Obesity.** Increased weight from younger to older ages is reported to be associated with statistically significant increased risk of breast cancer for pre- and post-menopausal women [49-53]. A study reported that obese (BMI at least 30 kg/m^2^) post-menopausal women have 31% excess risk compared to those whose BMI is less than 25 kg/m2 [54]. Moreover, post-menopausal obesity seems to be an important predictor of fatal breast cancer [55]. There are claims that there can be a protective effect on the development of cancer by maintaining normal BMI [56].

6. **Diet.** Studies report that high consumption of fruit and vegetables may offer significant protection against breast cancer [57-60]. Meat, especially red meat intake, is reported by some studies to be associated with increased breast cancer risk [61-63], though a few studies found no association [64,65]. A review of 13 prospective studies claim that higher fat intake does not confer excess risk of breast cancer [66]. Polyunsaturated fatty acids, especially from fish or shellfish, may offer protection, and saturated fat is likely to increase the risk of breast cancer [67-69]. However, data about other dietary factors have been inconsistent.

7. **Physical activity.** Increased total activity and recreational physical activity especially after menarche may decrease risk of breast cancer in postmenopausal women [70,71]. Between 75 and 150 minutes per week of brisk walking appears to decrease risk of breast cancer by 18% and strenuous physical activity at age 35 (rather than at 18 or 50 years) seems to result in a 14% risk reduction [71]. There seems to be a graded reduction in risk with increasing years of exercise [72]. A study reports that postmenopausal women with sedentary occupations have 49% higher risk of breast cancer than women in more physically demanding occupations [73].

8. **Smoking.** There is evidence that smoking especially during the period between menarche and first childbirth may increase the risk of breast cancer [74]. The increase in risk may be from 32% to 83% in smokers [75,76].

9. **Reproductive factors.** Overall risk of breast cancer in both pre- and post-menopausal women seems to increase with increasing age of first childbirth or first full term pregnancy, as well as with low parity and a nulliparous state [37,77]. Women with an age at first childbirth of 30 years or more are 5 to 7 times more likely to develop breast cancer compared with those aged less than 20 years [78,79]. Each additional birth may confer an average 10% reduction in breast cancer risk [80].

10. ** Severe lifetime events.** One study found that after adjustment for age, menopause and other potential confounders, severe life events increase the risk of breast cancer by 11 times [81].

11. ** Chemicals and radiation.** High exposure to polycyclic aromatic hydrocarbons may increase breast cancer risk by 50% [82]. Long term (more than 15 years) exposure to organic solvents, asbestos and vitreous fibres is likely to increase risk by 1.5 to 2 times [83]. Medium and high levels of occupational exposure to ionizing radiation, combined exposure to chest x-rays and occupational radiation as well as therapeutic radiation (e.g. for skin problems) are reported to increase breast cancer risk [84-86]. Exposure to sunlight may, on the other hand, be protective for breast cancer [87].

12. ** Healthy lifestyle index.** A recent case control study demonstrated that healthy lifestyle significantly reduces the odds of having breast cancer in both pre and post-menopausal women. Healthy lifestyle index was considered as the combined effect of moderate and/or vigorous-intensity physical activity, low consumption of fat, processed foods, refined cereals, complex sugars, and the avoidance of tobacco smoking and alcohol consumption [88].

### Findings from literature review about effective interventions in reducing the risk of breast cancer

Our final list of variables for inclusion into the audit-based education programme is those where change may reduce the risk of breast cancer; they include: weight control, dietary change, physical activity and reduction of alcohol intake. These are all variables readily extracted from primary care computer systems.

1. **Weight control.** Reduction in body weight is reported to reduce breast cancer risk, especially in postmenopausal women [49,89].

2. **Diet.** The evidence for effectiveness of dietary change in reducing breast cancer risk is relatively limited. A randomised trial in women with early stage breast cancer reported that a reduction in the amount of dietary fat of 18–19 g per day was associated with a decreased risk of breast cancer recurrence [90].

3. **Physical activity.** A minimum of 150 minutes and up to 420 minutes per week of moderate to vigorous intensity physical activity may decrease the risk of breast cancer [91].

4. **Alcohol intake reduction.** There is evidence that decreasing (by one drink or less per day) or stopping alcohol consumption altogether may reduce breast cancer risk in post-menopausal women [49,89].

### Practice profile and inter-practice variation

**Figure 1:**
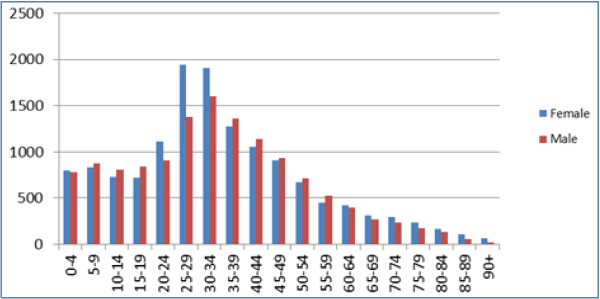
Age-sex profile of the combined practice populations.

**Figure 2:**
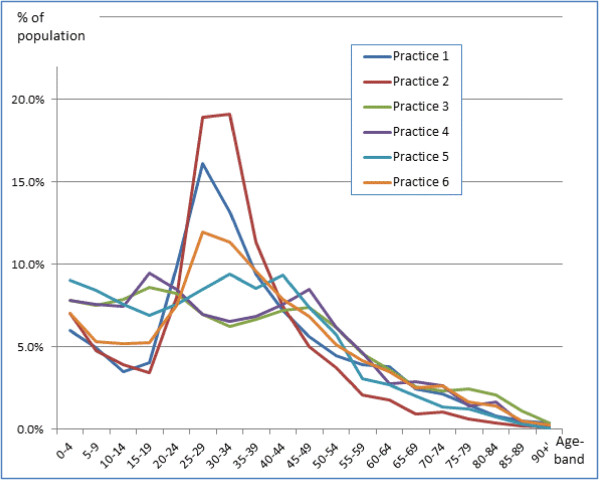
Age-band distribution by practice.

**Figure 3:**
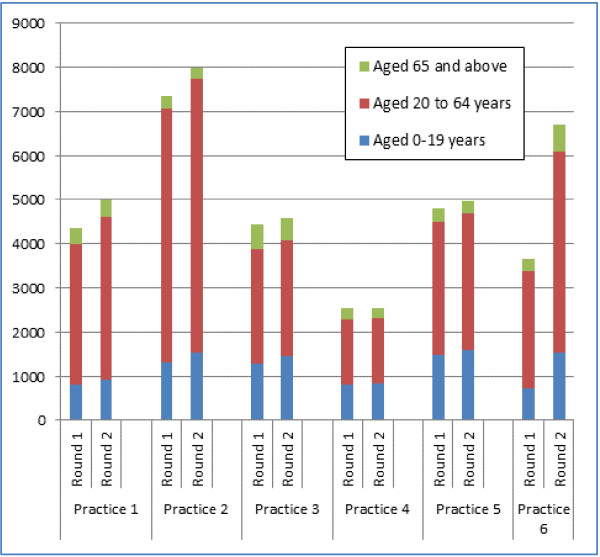
Age-bands at first and last data collections.

The practices were drawn from across London, they were largely in mixed areas with a social class distribution approximating to or just below the national average; two practices could be described as inner city, and one practice was located in a more affluent area. The practice population had a population younger than the national average, with an excess of younger women (Figure 1); The mean age recorded in the first round of data collection was 33.74 years while that recorded in the second round was 32.90 years (Additional file 1: Table S1). The practice populations had grown from a combined list size of 27,148 to 31,794 between the first and second data collections, though there was no statistical difference in the age-sex profile (Figures 2 and 3). Most of this growth occurred in practice 6 (out of the 6 practices taking part), which accounted for two-thirds of this growth (3,049/4,646 = 65.6%) (Additional file 1: Table S1). There was variation in the recording of data between practices before and after the intervention across the whole population (Chi-square p < 0.001, Table 1). No single practice had the highest level of recording in all domains.

**Table 1:** Variation in recording of key audit variables between practices for the first and second data collections in Adult Population (over 18 years)

**%**	**All family history**	**Family history of cancer**	**Alcohol consumption**	**Smoking status**	**Lifestyle advice**	**Practice population (n)**
**First data collection**						
Practice 1	33.6	2.8	89.2	97	75.8	3627
Practice 2	36.7	4.9	84.8	97	74.7	6158
Practice 3	34.4	7.4	65.3	96.5	74.7	3315
Practice 4	11.6	1.3	80.1	98.6	58.8	1841
Practice 5	57.5	10.2	73.8	95.6	81.4	3462
Practice 6	53	2.1	95.8	98.7	75	3000
**All**	**39.3**	**5.1**	**81.9**	**97.1**	**74.6**	**21403**
*Chi square*	p < 0.001	p < 0.001	p < 0.001	p < 0.001	p < 0.001	p < 0.001
**Second data collection**						
Practice 1	37.8	6.2	96.2	98.8	75.2	4161
Practice 2	40.5	8.1	89	98.1	77.7	6596
Practice 3	33.5	6.8	73	97.5	79.8	3285
Practice 4	13.6	2.5	80.6	99.2	50.7	1824
Practice 5	51.2	5.1	76.3	97.5	73.7	3512
Practice 6	45.7	1.9	90.8	97.2	60.5	5314
**All**	**39.8**	**5.4**	**86**	**97.9**	**71.3**	**24692**
*Chi square*	p < 0.001	p < 0.001	p < 0.001	p < 0.001	p < 0.001	p < 0.001

The proportions were different for all key variables.

### Pilot audit-based education intervention

The baseline data collection of lifestyle factors took place as planned with the exception of the technical problems reported above. The focus of the practices action plan was on improving quality of data recording at new patient medicals.

We extracted a dataset that would provide insight into whether the elements of a family history of breast cancer identified in the literature review were recorded. Only 1.3% of the female adult population had a history of breast or bowel cancer recorded. This increased to 1.8% in the after group, with this history being recorded for 2.8% of new patients (Table 2). Across the risk factors there was a large rise in the recording of information about family history of cancer in new patients; though overall FH recording was a little lower in new patients (40.6% compared with 43.0%). Alcohol consumption and smoking were consistently almost universally recorded; approximately three-quarters got lifestyle advice. New patients had both less contraceptive needs and pregnancies recorded; though had much higher requirements for oestrogens and hormone replacement therapy recorded.

**Table 2:** Rates of recording of data relevant to the assessment of a family history of breast cancer, in adult females (over 18 years)

**%**	**Practice population (N)**	**All family history**	**FH of neoplasm**	**FH of breast/bowel cancer**	**Alcohol intake**	**Smoking status**	**Lifestyle advice**	**Oral contraception**	**Oestrogens/HRT**	**Pregnancy ever**
**Baseline**										
Practice 1	2105	36.4	3.8	0.9	92.4	98.8	78.9	14.9	5.9	19.5
Practice 2	3193	40.1	6.8	1.8	89.1	98.8	77.6	8.6	3.7	20
Practice 3	1646	37.7	8.6	2.3	72.2	98.1	73.9	9.2	13.7	22.4
Practice 4	908	14.5	1.5	0.7	87.3	99.8	64.3	5.9	14.1	21.5
Practice 5	1687	60.8	13.3	0.7	75.9	96.7	84.8	10.3	9.4	33.1
Practice 6	1658	56.9	2.4	1.1	98.3	99.5	77.6	6.1	2.2	15.8
All	11197	42.6	6.4	1.3	86.5	98.6	77.3	9.5	7.1	21.7
**Post Audit**										
Practice 1	2481	41.8	7.7	2.5	97.9	99.6	76.1	20.8	10	15.9
Practice 2	3403	45.3	11	2.7	92	99.4	79.1	13.4	5.3	20
Practice 3	1644	36.6	7.8	1.9	80.1	99	79	14.7	18.3	22.6
Practice 4	911	15.9	2.7	0.9	87.4	99.9	53.9	8	21.3	22.5
Practice 5	1684	54.9	6.9	0.7	78.1	98.8	75.9	13.8	13.8	31.4
Practice 6	2839	46.6	2.3	1	93.3	98.6	59.5	8.3	0	19.6
**All**	**12962**	**43.0**	**6.9**	**1.8**	**89.8**	**99.2**	**72**	**13.5**	**8.9**	**21.1**
**New patients**										
Practice 1	744	47.7	15.6	4.7	98.9	98.9	80	16.5	1.7	7.9
Practice 2	877	50.7	21.2	3.6	92.8	99.2	91.1	9.5	2.3	11.5
Practice 3	173	28.3	6.9	0.6	93.6	97.7	20.8	4.6	2.3	19.7
Practice 4	86	29.1	12.8	1.2	72.1	100	36	3.5	8.1	19.8
Practice 5	209	11.5	0.5	0	86.6	94.7	42.6	3.3	3.8	16.7
Practice 6	583	32.1	3.6	1	91.9	97.4	46.3	6.5	0	11.1
**All**	**2672**	**40.6**	**13.0**	**2.8**	**93.2**	**98.3**	**68.1**	**9.8**	**19**	**11.6**

**Figure 4:**
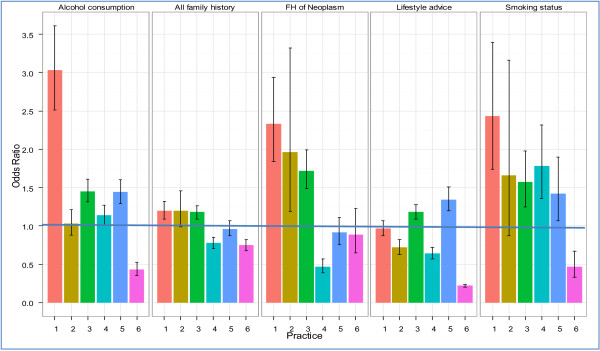
**Odds ratios (OR) and 95% Confidence Intervals (CI) for a change in data recording between the first (baseline) and second data collections.** An OR >1 suggests that data recording is more likely after the second data collection. Only practice 3 had the OR of recording all data domains increased after the intervention than before.

After the educational intervention, there was an increase in the rate of recording in all categories of data other than ‘Life-style advice/education’ (Table 3). However, the 95% confidence intervals for family history and family history of cancer data recording were not statistically significant; the Odds Ratio (OR) was 1.02 (95% CI 0.98 to 1.06); and 1.08 (95% CI 0.99 to 1.17) respectively. There was a statistically significant, 36% increase in the recording of alcohol consumption; (95% CI 1.30 to 1.43); and 42% increase for smoking; (1.42, 95% CI 1.27 to 1.60). There was a fall of 16% in the recording of lifestyle advice given which was also significant; the OR was 0.84 (95% CI 0.81-0.88). The changes by practices are shown in Figure 4.

**Table 3:** Odds ratios (OR) and 95% Confidence Intervals (CI) for a change in data recording between the first (baseline) and second data collections

	**All family history**	**FH of neoplasm**	**Alcohol consumption**	**Smoking status**	**Lifestyle advice**
**Practice 1**	1.20	2.33	3.03	2.43	0.97
OR (95% CI)	(1.09 to 1.32)*	(1.84 to 2.94)*	(2.51 to 3.61)*	(1.74 to 3.39)*	(0.87 to 1.07)
**Practice 3**	1.18	1.72	1.45	1.57	1.18
OR (95% CI)	(1.09 to 1.26)*	(1.49 to 1.99)*	(1.31 to 1.61)*	(1.25 to 1.98)*	(1.09 to 1.28)*
**Practice 5**	0.96	0.92	1.44	1.42	1.34
OR (95% CI)	(0.87 to 1.07)	(0.76 to 1.11)	(1.29 to 1.60)*	(1.07 to 1.90)*	(1.20 to 1.51)*
**Practice 2**	1.20	1.96	1.03	1.66	0.72
OR (95% CI)	(0.99 to 1.46)	(1.19 to 3.32)*	(0.88 to 1.21)	(0.87 to 3.16)	(0.63 to 0.82)
**Practice 4**	0.78	0.47	1.14	1.78	0.64
OR (95% CI)	(0.71 to 0.85)	(0.39 to 0.57)	(1.02 to 1.27)*	(1.36 to 2.32)*	(0.57 to 0.72)
**Practice 6**	0.75	0.89	0.43	0.47	0.22
OR (95% CI)	(0.68 to 0.82)	(0.65 to 1.2294)	(0.35 to 0.53)	(0.33 to 0.67)	(0.20 to 0.24)
**All**	1.02	1.08	1.36	1.42	0.84
**OR (95%CI)**	(0.98 to 1.06)	(0.99 to 1.17)	(1.30 to 1.43)*	(1.27 to 1.60)*	(0.81 to 0.88)

An OR >1 suggests that data recording is more likely after the second data collection.

*statistically significant change.

### Qualitative findings – feedback from the workshop

The practices we approached readily agreed to participate, and saw value in improving their data, and participating in the audit. Qualitative feedback at the workshop highlighted how both practitioners recorded and computerised medical record systems were inconsistent in how family history was recorded; and stated that when new patients register is the best time to improve recording. Discussions with practitioners, at the time of the baseline data workshop and presentation revealed inconsistent use of codes, others using high level non-specific codes (e.g. family history of cancer) rather than disease specific codes. In addition, there was very little use of negative codes (e.g. no family history of breast cancer.) The computerised records factors included reporting differences in where in the system family history was recorded, inconsistency in the information requested (for example some systems asked which family member), and inconsistencies in the coding system (for example there are codes for a negative family history of breast cancer, whereas there are for no family history of bowel and ovarian cancer; 122 f. and 122G. respectively)). There are also family history codes in different parts of the coding hierarchy; practitioners did not like the codes that displayed an asterisk (i.e. The family history of neoplasm, 124. codes, display as FH *. The specific code for “FH*-breast” is 1243. They reported these as potentially paternalistic; it felt to practitioners wrong that explicit records of a family history of cancer were data that could not be displayed as such on the computer screen and hence be readily shared with the patient.

## Discussion

### Principal findings

Whilst overall the participants in the audit and feedback intervention improved the quality of their data after the audit, there was considerable variation reported between practices and practitioners; and though some improved their coding during the audit period, others did not. We found that a wide range of different codes were being used to represent the same clinical problem, some practitioners coded in a highly specific way while others were much less specific using high level codes. The practitioners who participated in our audit placed their emphasis on improving the quality of recording after doing new patient medical examinations; this may lead to the anticipated rate of improvement in data quality to the rate of turnover or growth of the practices when new patients are registered.

### Implications of the findings

Although general practice is encouraged to record family history there are no national standards that need to be adhered to; this should be corrected. Pilot practices appeared willing to engage in this initiative and we hope that a broader group of practices would want to engage in this type of quality improvement. Although we did achieve some progress, the rate of progress was slow. Moreover, despite our assumption that improved data recording might be found in new patients, we would hope that existing patients might also benefit from this intervention. Raising awareness about good quality recording would steadily improve recording and data quality over time. We should consider building on this, maybe creating a preferred list of codes that could appear in a data entry form, or template, to facilitate the consistent collection of data which has been collected in this audit. A recommended code list could facilitate data entry and the audit of quality; and ultimately the quality of care.

### Limitations of the method

Integrated risk assessment tools for cancer are not embedded within primary care computer records. We did not explore whether integrated tools would be a greater stimulus to data recording and risk assessment. Such systems would raise awareness around familial breast cancer and then allow direct integration of family history into electronic GP records.

The data we looked at were those related to reducing cancer risk. Recording of these data *per se* does not mean that a risk assessment has been carried out. We made the same assumptions as when we have critically appraised diabetes [22,23]; namely that data recording approximates to the clinician’s engagement with the care process whilst recognising that computer data can under or over represent the actual care provided to an individual patient.

There were gaps in the data collection, particularly BMI data; however this aspect of the project is probably the easiest to put right.

We did not take into account women’s views of how they perceive risk, the current services, or where they see gaps in provision [92-94].

It is recognised that good quality recording of family history is a neglected theme in providing care, and more could be done in primary care [95,96]. Other studies have also found recording of family history of breast cancer to be limited in terms of recording [97].

The theory of diffusion of innovation may provide insights into how change in data recording may be adopted [98]. This theory describes the very slow process whereby innovators take on a new process, followed by early adopters, and followed in sequence by other groups. However diffusion can be a slow process, taking many years – an intervention like audit based education might accelerate uptake of guidance. Other initiatives have required the setting of a recommended coding list to encourage consistency of coding [99]. Involvement of the appropriate primary care society and support from cancer charities, professional associations and academies may also facilitate the uptake of good and accurate data recording. Since initial risk assessment of a new patient is a crucial component which takes place at new patient medicals, then practice nurses will also need appropriate education [100].

Inconsistencies in family history recording were likely to be related to data recording rather than data extraction. Other than our queries having a code error for BMI, the data collectors did not report any problems with data extraction, we have a set format for reporting these [92]. In the qualitative results section we explained how personal practitioner preference for using either generic or specific codes, and the nature of the coding system was responsible for variation in the prevalence and granularity of the coding used. In addition, variation in picking-lists (the lists practitioners select codes from) and different ways that computerised medical record system vendors create these lists tends to foster and perpetuate differences in coding between practices [101]. The creation of limited lists of codes for pay-for-performance that has driven standardisation of coding [102]. We have demonstrated some success with a similar approach, a recommended limited list of codes, for the recording child safeguarding issues in primary care; where flagging issues can help with stratifying risks in later consultations [103].

### Call for further research

A consensus building exercise is needed to offer preferred coding strategies. Risk stratifying software should be piloted and compared with ABE and usual practice, as a tool for conducting risk assessments. Looking at rates of appropriate referral for breast assessment would be one measure. Measuring the need for genetic counselling or onward referral to genetics centres could serve as another outcome measure. The opportunity now exists for building on what has been learned from previous trials, which took place prior to the widespread use of computers to support chronic disease management [104,105]. Strategies such as providing online risk assessment tools, directly to patients, could be considered, however a recent trial has indicated that such online tools may have limited impact [106].

## Conclusions

We implemented audit-based education (ABE) in six practices; and it has provided insight into the level of data recording, and that improved assessment of family history and other risk factors is most easily improved at the time of new patient appointments. However, more needs to be done to improve assessment across practice populations.

There was no significant change in the recording of family history of disease or cancer after the audit, though the recording of risk factors improved.

The study provided insight into variation and issues with current data recording, the willingness of practitioners to collaborate and rate of change achieved by audit and feedback. Whilst recording of risk factors improved with audit-based education further interventions are needed to raise data quality and care to standards where people with a small increased risk are adequately flagged in primary care records.

## Competing interests

The authors declare that they have no competing interests.

## Authors’ contributions

IR conceived the audit design with SdeL, secured the funding, presented the intervention, wrote the first draft document and responded to further drafts. SC carried out the breast cancer risk literature review and contributed to all drafts of the paper. TC carried out the data analysis and was involved in the writing of the early draft documents and contributed to all drafts of the paper. IJ developed the final paper and assisted with the revision of the final paper. AT recruited practices to take part in the audit and contributed to all drafts of the paper. SdeL conceived the audit design with IR., worked with colleagues to develop the audit package, led the informatics aspects of the study, wrote the first version of the complete paper from draft documents supplied by IR, SC, and TC, and contributed to all drafts of the paper. SdeL also wrote the first revision post peer review. All authors read and approved the final manuscript.

## Pre-publication history

The pre-publication history for this paper can be accessed here:

http://www.biomedcentral.com/1471-2296/14/105/prepub

## Supplementary Material

Additional file 1 **Box S1.** Epidemiology of breast cancer in the UK. Box S2. Audit-based education (ABE) – a quality improvement method. The quality improvement strategy developed by the Primary Care Data Quality Programme (PCDQ). Table S1. Age-sex profiles of participating practices. Table S2. Growth in practice populations between first and second data collections. Table S3. Availability of key data fields in first and second data collections. Click here for file

## References

[B1] Parkin DM Boyd L Walker LC The fraction of cancer attributable to lifestyle and environmental factors in the UK in 2010 Summary and conclusions 2011 105 S2 S77 S81 10.1038/bjc.2011.489 PMC3252065 22158327

[B2] Key TJ Verkasalo Banks PK Epidemiology of breast cancer Lancet Oncol 2001 2 3 133 140 10.1016/S1470-2045(00)00254-0 11902563

[B3] Cancer Research UK Breast Cancer Incidence Statistics[http://www.cancerresearchuk.org/cancer-info/cancerstats/types/breast/incidence/uk-breast-cancer-incidence-statistics]

[B4] National Institute for Health and Clinical Excellence (NICE) Familial breast cancer: The classification and care of women at risk of familial breast cancer in primary, secondary and tertiary care. CG41. October 2006 **[**http://www.nice.org.uk/CG41]

[B5] Department of Health Publications and Policy Guidance. Cancer reform strategy [http://www.dh.gov.uk/en/Publicationsandstatistics/Publications/PublicationsPolicyAndGuidance/DH_081006]

[B6] Rubin G McPhail S Elliott K National Audit of Cancer Diagnosis in Primary Care. London; Royal College of General Practitioners, 2011 [http://www.dur.ac.uk/resources/school.health/erdu/NationalAuditofCancerDiagnosisinPrimaryCare.pdf]

[B7] Johnson N Lancaster T Fuller A Hodgson SV The prevalence of a family history of cancer in general practice Fam Pract 1995 12 3 287 289 10.1093/fampra/12.3.287 8536831

[B8] Silva E Genetic counseling and clinical management of newly diagnosed breast cancer patients at genetic risk for BRCA germline mutations: perspective of a surgical oncologist Fam Cancer 2008 7 1 91 95 10.1007/s10689-007-9167-3 17943460

[B9] Teams without walls The value of medical innovation and leadership. Report of a working Party of the RCP, RCGP and RCPCH. 2008 [http://www.rcpch.ac.uk/sites/default/files/teams-without-walls.pdf.pdf]

[B10] de Lusignan S Van Weel C The use of routinely collected computer data for research in primary care: opportunities and challenges Fam Pract 2006 23 2 253 263 16368704 10.1093/fampra/cmi106

[B11] Schade CP Sullivan FM De Lusignan S Madeley J e-Prescribing, efficiency, quality: lessons from the computerization of UK family practice J Am Med Inform Assoc 2006 13 5 470 475 10.1197/jamia.M2041 16799129 PMC1561797

[B12] de Lusignan S Chan T The development of primary care information technology in the United kingdom J Ambul Care Manage 2008 31 3 201 210 10.1097/01.JAC.0000324664.88131.d2 18574377

[B13] de Lusignan S An educational intervention, involving feedback of routinely collected computer data, to improve cardiovascular disease management in UK primary care Methods Inf Med 2007 46 1 57 62 17224982

[B14] de Lusignan S Hague N Brown A Majeed A An educational intervention to improve data recording in the management of ischaemic heart disease in primary care J Public Health (Oxf) 2004 26 1 34 37 10.1093/pubmed/fdh104 15044571

[B15] de Lusignan S Belsey J Hague N Dhoul N Van Vlymen J Audit-based education to reduce suboptimal management of cholesterol in primary care: a before and after study J Public Health (Oxf) 2006 28 4 361 369 10.1093/pubmed/fdl052 17038329

[B16] de Lusignan S Gallagher H Chan T Thomas N Van Vlymen J Nation M Jain N Tahir A Du Bois E Crinson I Hague N Reid F Harris K The QICKD study protocol: a cluster randomised trial to compare quality improvement interventions to lower systolic BP in chronic kidney disease (CKD) in primary care Implement Sci 2009 14 4 39 19602233 10.1186/1748-5908-4-39 PMC2719588

[B17] de Lusignan S Gallagher H Jones S Chan T Van Vlymen J Tahir A Thomas N Jain N Dmitrieva O Rafi I McGovern A Harris K Audit-based education lowers systolic blood pressure in chronic kidney disease: the Quality Improvement in CKD (QICKD) trial results Kidney Int 2013 [Epub ahead of print] 10.1038/ki.2013.96 PMC3778715 23536132

[B18] Forsetlund L Bjørndal A Rashidian A Jamtvedt G O’Brien MA Wolf F Davis D Odgaard-Jensen J Oxman AD Continuing education meetings and workshops: effects on professional practice and health care outcomes Cochrane Database Syst Rev 2009 2 CD003030 10.1002/14651858.CD003030.pub2 19370580 PMC7138253

[B19] Jamtvedt G Young JM Kristoffersen DT O’Brien MA Oxman AD Audit and feedback: effects on professional practice and health care outcomes Cochrane Database Syst Rev 2012 6 CD000259 10.1002/14651858.CD000259.pub3 12917891

[B20] Hysong SJ Meta-analysis: audit and feedback features impact effectiveness on care quality Med Care 2009 47 3 356 363 10.1097/MLR.0b013e3181893f6b 19194332 PMC4170834

[B21] Van Vlymen J de Lusignan S Hague N Chan T Dzregah B Ensuring the Quality of Aggregated General Practice Data: Lessons from the Primary Care Data Quality Programme (PCDQ) Stud Health Technol Inform 2005 116 1010 1015 16160391

[B22] de Lusignan S Khunti K Belsey J Hattersley A Van Vlymen J Gallagher H Millett C Hague NJ Tomson C Harris K Majeed A A method of identifying and correcting miscoding, misclassification and misdiagnosis in diabetes: a pilot and validation study of routinely collected data Diabet Med 2010 27 2 203 209 10.1111/j.1464-5491.2009.02917.x 20546265

[B23] de Lusignan S Sadek N Mulnier H Tahir A Russell-Jones D Khunti K Miscoding, misclassification and misdiagnosis of diabetes in primary care Diabet Med 2012 29 2 181 189 10.1111/j.1464-5491.2011.03419.x 21883428

[B24] Altman DG Practical statistics for medical research 1990 London: Chapman & Hall 234 235

[B25] National Patient Safety Agency (NPSA) National Research Ethics Service (NRES) Defining Research NRES guidance to help you decide if your project requires review by a Research Ethics Committee Ref: 0987 December 2009. [http://www.hpa.org.uk/webc/HPAwebFile/HPAweb_C/1272032326180]

[B26] European society of human reproduction and embryology Hormones and breast cancer Hum Reprod Update 2004 10 4 281 293 15192054 10.1093/humupd/dmh025

[B27] Collaborative group on hormonal factors in breast cancer Breast cancer and hormone replacement therapy: collaborative reanalysis of data from 51 epidemiological studies of 52,705 women with breast cancer and 108,411 women without breast cancer Lancet 1997 350 9084 1047 1059 10213546

[B28] Dumeaux V Alsaker E Lund E Breast cancer and specific types of oral contraceptives: a large Norwegian cohort study Int J Cancer 2003 105 844 850 10.1002/ijc.11167 12767072

[B29] Althuis MD Brogan DR Coates RJ Daling JR Gammon MD Malone KE Schoenberg JB Brinton LA Hormonal content and potency of oral contraceptives and breast cancer risk among young women Br J Cancer 2003 88 1 50 57 10.1038/sj.bjc.6600691 12556959 PMC2376784

[B30] Chen WY Hankinson SE Schnitt SJ Rosner BA Holmes MD Colditz GA Association of hormone replacement therapy to estrogen and progesterone receptor status in invasive breast carcinoma Cancer 2004 101 7 1490 1500 10.1002/cncr.20499 15378477

[B31] Beral V Breast cancer and hormone-replacement therapy in the million women study Lancet 2003 362 9382 419 427 12927427 10.1016/s0140-6736(03)14065-2

[B32] Milne RL Knight JA John EM Dite GS Balbuena R Ziogas A Andrulis IL West DW Li FP Southey MC Giles GG McCredie MR Hopper JL Whittemore AS Oral contraceptive use and risk of early-onset breast cancer in carriers and noncarriers of BRCA1 and BRCA2 mutations Cancer Epidemiol Biomarkers Prev 2005 14 2 350 356 10.1158/1055-9965.EPI-04-0376 15734957

[B33] Jernstrom H Bendahl PO Lidfeldt J Nerbrand C Agardh CD Samsioe G A prospective study of different types of hormone replacement therapy use and the risk of subsequent breast cancer: the women’s health in the lund area (WHILA) study (Sweden) Cancer Causes Control 2003 14 7 673 680 10.1023/A:1025635720208 14575365

[B34] Newcomb PA Titus-Ernstoff L Egan KM Trentham-Dietz A Baron JA Storer BE Willett WC Stampfer MJ Postmenopausal estrogen and progestin use in relation to breast cancer risk Cancer Epidemiol Biomarkers Prev 2002 11 593 600 12101105

[B35] Pitkin J Rees MC Gray S Lumsden MA Marsden J Stevenson J Williamson J; British Menopause Society Council: **Managing the menopause: British Menopause Society Council consensus statement on hormone replacement therapy** J Br Menopause Soc 2005 11 4 152 156 10.1258/136218005775544354 16354459

[B36] Kellen E Vansant G Christiaens MR Neven P Van LE Lifestyle changes and breast cancer prognosis: a review Breast Cancer Res Treat 2009 114 1 13 22 10.1007/s10549-008-9990-8 18389367

[B37] Rieck G Fiander A The effect of lifestyle factors on gynaecological cancer Best Pract Res Clin Obstet Gynaecol 2006 20 2 227 251 10.1016/j.bpobgyn.2005.10.010 16543119

[B38] Lee SY Kim MT Kim SW Song MS Yoon SJ Effect of lifetime lactation on breast cancer risk: a Korean women’s cohort study Int J Cancer 2003 105 390 393 10.1002/ijc.11078 12704674

[B39] Helewa M Levesque P Provencher D Lea RH Rosolowich V Shapiro HM; Breast Disease Committee and Executive Committeee and Council; Society of Obstetricians and Gynaecologists of Canada Breast cancer, pregnancy, and breastfeeding J Obstet Gynaecol Can 2002 24 164 180 12196882

[B40] Key J Hodgson S Omar RZ Jensen TK Thompson SG Boobis AR Davies DS Elliott P Meta-analysis of studies of alcohol and breast cancer with consideration of the methodological issues Cancer Causes Control 2006 17 6 759 770 10.1007/s10552-006-0011-0 16783604

[B41] Tjønneland A Christensen J Olsen A Stripp C Thomsen BL Overvad K Peeters PH Van Gils CH Bueno-de-Mesquita HB Ocké MC Thiebaut A Fournier A Clavel-Chapelon F Berrino F Palli D Tumino R Panico S Vineis P Agudo A Ardanaz E Martinez-Garcia C Amiano P Navarro C Quirós JR Key TJ Reeves G Khaw KT Bingham S Trichopoulou A Trichopoulos D Naska A Nagel G Chang-Claude J Boeing H Lahmann PH Manjer J Wirfält E Hallmans G Johansson I Lund E Skeie G Hjartåker A Ferrari P Slimani N Kaaks R Riboli E Alcohol intake and breast cancer risk: the European Prospective Investigation into Cancer and Nutrition (EPIC) Cancer Causes Control 2007 18 4 361 373 10.1007/s10552-006-0112-9 17364225

[B42] Morch LS Johansen D Thygesen LC Tjønneland A Løkkegaard E Stahlberg C Grønbaek M Alcohol drinking, consumption patterns and breast cancer among Danish nurses: a cohort study Eur J Public Health 2007 17 6 624 629 10.1093/eurpub/ckm036 17442702

[B43] Zhang SM Lee IM Manson JE Cook NR Willett WC Buring JE Alcohol consumption and breast cancer risk in the Women’s Health Study Am J Epidemiol 2007 165 6 667 676 10.1093/aje/kwk054 17204515

[B44] Visvanathan K Crum RM Strickland PT You X Ruczinski I Berndt SI Alberg AJ Hoffman SC Comstock GW Bell DA Helzlsouer KJ Alcohol dehydrogenase genetic polymorphisms, low-to-moderate alcohol consumption, and risk of breast cancer Alcohol Clin Exp Res 2007 31 3 467 476 10.1111/j.1530-0277.2006.00334.x 17295732 PMC2787101

[B45] Harvey EB Schairer C Brinton LA Hoover RN Fraumeni JF Jr Alcohol consumption and breast cancer J Natl Cancer Inst 1987 78 657 661 3104648

[B46] Manisto S Virtanen M Kataja V Uusitupa M Pietinen P Lifetime alcohol consumption and breast cancer: a case–control study in Finland Public Health Nutr 2000 3 11 18 10786719 10.1017/s1368980000000033

[B47] Zhang Y Kreger BE Dorgan JF Splansky GL Cupples LA Ellison RC Alcohol consumption and risk of breast cancer: the Framingham study revisited Am J Epidemiol 1999 149 102 104 10.1093/oxfordjournals.aje.a009773 9921953

[B48] Singletary SE Rating the risk factors for breast cancer Ann Surg 2003 237 4 474 482 12677142 10.1097/01.SLA.0000059969.64262.87 PMC1514477

[B49] Eliassen AH Colditz GA Rosner B Willett WC Hankinson SE Adult weight change and risk of postmenopausal breast cancer JAMA 2006 296 2 193 201 10.1001/jama.296.2.193 16835425

[B50] Harvie M Howell A Vierkant RA Kumar N Cerhan JR Kelemen LE Folsom AR Sellers TA Association of gain and loss of weight before and after menopause with risk of postmenopausal breast cancer in the Iowa women’s health study Cancer Epidemiol Biomarkers Prev 2005 14 3 656 661 10.1158/1055-9965.EPI-04-0001 15767346

[B51] Lahmann PH Schulz M Hoffmann K Boeing H Tjønneland A Olsen A Overvad K Key TJ Allen NE Khaw KT Bingham S Berglund G Wirfält E Berrino F Krogh V Trichopoulou A Lagiou P Trichopoulos D Kaaks R Riboli E Long-term weight change and breast cancer risk: the European Prospective Investigation into Cancer and Nutrition (EPIC) Br J Cancer 2005 93 5 582 589 10.1038/sj.bjc.6602763 16136032 PMC2361598

[B52] Feigelson HS Jonas CR Teras LR Thun MJ Calle EE Weight gain, body mass index, hormone replacement therapy, and postmenopausal breast cancer in a large prospective study Cancer Epidemiol Biomarkers Prev 2004 13 2 220 224 10.1158/1055-9965.EPI-03-0301 14973094

[B53] Radimer KL Ballard-Barbash R Miller JS Fay MP Schatzkin A Troiano R Kreger BE Splansky GL Weight change and the risk of late-onset breast cancer in the original Framingham cohort Nutr Cancer 2004 49 1 7 13 10.1207/s15327914nc4901_2 15456630

[B54] Lahmann PH Hoffmann K Allen N Van Gils CH Khaw KT Tehard B Berrino F Tjønneland A Bigaard J Olsen A Overvad K Clavel-Chapelon F Nagel G Boeing H Trichopoulos D Economou G Bellos G Palli D Tumino R Panico S Sacerdote C Krogh V Peeters PH Bueno-de-Mesquita HB Lund E Ardanaz E Amiano P Pera G Quirós JR Martínez C Tormo MJ Wirfält E Berglund G Hallmans G Key TJ Reeves G Bingham S Norat T Biessy C Kaaks R Riboli E Body size and breast cancer risk: findings from the European prospective investigation into cancer and nutrition (EPIC) Int J Cancer 2004 111 5 762 771 10.1002/ijc.20315 15252848

[B55] CuiY WMK Flaws JA Langenberg PTKH Bush TL Body mass and stage of breast cancer at diagnosis Int J Cancer 2002 98 279 283 10.1002/ijc.10209 11857420

[B56] Vainio H Kaaks R Bianchini F Weight control and physical activity in cancer prevention: international evaluation of the evidence Eur J Cancer Prev 2002 11 Suppl 2 S94 S100 12570341

[B57] Smith-Warner SA Spiegelman D Yaun SS Adami HO Beeson WL Van Den Brandt PA Folsom AR Fraser GE Freudenheim JL Goldbohm RA Graham S Miller AB Potter JD Rohan TE Speizer FE Toniolo P Willett WC Wolk A Zeleniuch-Jacquotte A Hunter DJ Intake of fruits and vegetables and risk of breast cancer: a pooled analysis of cohort studies JAMA 2001 285 769 776 10.1001/jama.285.6.769 11176915

[B58] Van Gils CH Peeters PH Bueno-de-Mesquita HB Boshuizen HC Lahmann PH Clavel-Chapelon F Thiébaut A Kesse E Sieri S Palli D Tumino R Panico S Vineis P Gonzalez CA Ardanaz E Sánchez MJ Amiano P Navarro C Quirós JR Key TJ Allen N Khaw KT Bingham SA Psaltopoulou T Koliva M Trichopoulou A Nagel G Linseisen J Boeing H Berglund G Wirfält E Hallmans G Lenner P Overvad K Tjønneland A Olsen A Lund E Engeset D Alsaker E Norat T Kaaks R Slimani N Riboli E Consumption of vegetables and fruits and risk of breast cancer JAMA 2005 293 2 183 193 10.1001/jama.293.2.183 15644545

[B59] Hirose K Takezaki T Hamajima N Miura S Tajima K Dietary factors protective against breast cancer in Japanese premenopausal and postmenopausal women Int J Cancer 2003 107 276 282 10.1002/ijc.11373 12949807

[B60] Adzersen KH Jess P Freivogel KW Gerhard I Bastert G Raw and cooked vegetables, fruits, selected micronutrients, and breast cancer risk: a case–control study in Germany Nutr Cancer 2003 46 131 137 10.1207/S15327914NC4602_05 14690788

[B61] Shannon J Cook LS Stanford JL Dietary intake and risk of postmenopausal breast cancer (United States) Cancer Causes Control 2003 14 19 27 10.1023/A:1022506507984 12708721

[B62] Tavani A La Vecchia C Gallus S Lagiou P Trichopoulos D Levi F Negri E Red meat intake and cancer risk: a study in Italy Int J Cancer 2000 86 425 428 10.1002/(SICI)1097-0215(20000501)86:3<425::AID-IJC19>3.0.CO;2-S 10760833

[B63] Hebert JR Hurley TG Ma Y The effect of dietary exposures on recurrence and mortality in early stage breast cancer Breast Cancer Res Treat 1998 51 17 28 10.1023/A:1006056915001 9877026

[B64] Missmer SA Smith-Warner SA Spiegelman D Yaun SS Adami HO Beeson WL Van Den Brandt PA Fraser GE Freudenheim JL Goldbohm RA Graham S Kushi LH Miller AB Potter JD Rohan TE Speizer FE Toniolo P Willett WC Wolk A Zeleniuch-Jacquotte A Hunter DJ Meat and dairy food consumption and breast cancer: a pooled analysis of cohort studies Int J Epidemiol 2002 31 78 85 10.1093/ije/31.1.78 11914299

[B65] Potischman N Coates RJ Swanson CA Carroll RJ Daling JR Brogan DR Gammon MD Midthune D Curtin J Brinton LA Increased risk of early-stage breast cancer related to consumption of sweet foods among women less than age 45 in the United States Cancer Causes Control 2002 13 937 946 10.1023/A:1021919416101 12588090

[B66] Hanf V Gonder U Nutrition and primary prevention of breast cancer: foods, nutrients and breast cancer risk Eur J Obstet Gynecol Reprod Biol 2005 123 2 139 149 10.1016/j.ejogrb.2005.05.011 16316809

[B67] Gago-Dominguez M Yuan JM Lee CL Yu MC HP Opposing effects of dietary n-3 and n-6 fatty acids on mammary carcinogenesis. The Singapore Chinese Health Study Br J Cancer 2003 89 1686 1692 10.1038/sj.bjc.6601340 14583770 PMC2394424

[B68] Richter WO Fatty acids and breast cancer-is there a relationship? Eur J Med Res 2003 8 373 380 12915333

[B69] Rissanen H Knekt P Jarvinen R Salminen I Hakulinen T Serum fatty acids and breast cancer incidence Nutr Cancer 2003 45 168 175 10.1207/S15327914NC4502_05 12881010

[B70] Carpenter CL Ross RK Paganini-Hill A Bernstein L Effect of family history, obesity and exercise on breast cancer risk among postmenopausal women Int J Cancer 2003 106 1 96 102 10.1002/ijc.11186 12794763

[B71] McTiernan A Kooperberg C White E Wilcox S Coates R Adams-Campbell LL Woods N Ockene J Women’s Health Initiative Cohort Study Recreational physical activity and the risk of breast cancer in postmenopausal women: The Women’s Health Initiative Cohort Study JAMA 2003 290 1331 1336 10.1001/jama.290.10.1331 12966124

[B72] Matthews CE Shu XO Jin F Dai Q Hebert JR Ruan ZX Gao YT Zheng W Lifetime physical activity and breast cancer risk in the Shanghai Breast Cancer Study Br J Cancer 2001 84 994 1001 10.1054/bjoc.2000.1671 11286483 PMC2363839

[B73] Moradi T Nyren O Zack M Magnusson C Persson I Adami HO Breast cancer risk and lifetime leisure-time and occupational physical activity (Sweden) Cancer Causes Control 2000 11 6 523 531 10.1023/A:1008900512471 10880034

[B74] Ha M Mabuchi K Sigurdson AJ Freedman DM Linet MS Doody MM Hauptmann M Smoking cigarettes before first childbirth and risk of breast cancer Am J Epidemiol 2007 166 55 61 10.1093/aje/kwm045 17426039

[B75] Reynolds P Hurley S Goldberg DE Anton-Culver H Bernstein L Deapen D Horn-Ross PL Peel D Pinder R Ross RK West D Wright WE Ziogas A Active smoking, household passive smoking, and breast cancer: evidence from the California teachers study J Natl Cancer Inst 2004 96 29 37 10.1093/jnci/djh002 14709736

[B76] Terry PD Miller AB Rohan TE Cigarette smoking and breast cancer risk: a long latency period? Int J Cancer 2002 100 723 728 10.1002/ijc.10536 12209614

[B77] Althuis MD Fergenbaum JH Garcia-Closas M Brinton LA Madigan MP Sherman ME Etiology of hormone receptor-defined breast cancer: a systematic review of the literature Cancer Epidemiol Biomarkers Prev 2004 13 10 1558 1568 15466970

[B78] Tavani A Gallus S La Vecchia C Negri E Montella M Dal Maso L Franceschi S Risk factors for breast cancer in women under 40 years Eur J Cancer 1999 35 1361 1367 10.1016/S0959-8049(99)00139-2 10658528

[B79] Tryggvadottir L Tulinius H Eyfjord JE Sigurvinsson T Breast cancer risk factors and age at diagnosis: an Icelandic cohort study Int J Cancer 2002 98 604 608 10.1002/ijc.10217 11920622

[B80] Lambe M Hsieh CC Chan HW Ekbom A Trichopoulos D Adami HO Parity, age at first and last birth, and risk of breast cancer: a population-based study in Sweden Breast Cancer Res Treat 1996 38 305 311 10.1007/BF01806150 8739084

[B81] Chen CC David AS Nunnerley H Michell M Dawson JL Berry H Dobbs J Fahy T Adverse life events and breast cancer: case–control study Br Med J 1995 311 7019 1527 1530 10.1136/bmj.311.7019.1527 8520393 PMC2548223

[B82] Gammon MD Santella RM Neugut AI Eng SM Teitelbaum SL Paykin A Levin B Terry MB Young TL Wang LW Wang Q Britton JA Wolff MS Stellman SD Hatch M Kabat GC Senie R Garbowski G Maffeo C Montalvan P Berkowitz G Kemeny M Citron M Schnabel F Schuss A Hajdu S Vinceguerra V Environmental toxins and breast cancer on Long Island. I. Polycyclic aromatic hydrocarbon DNA adducts Cancer Epidemiol Biomarkers Prev 2002 11 677 685 12163319

[B83] Laden F Collman G Iwamoto K Alberg AJ Berkowitz GS Freudenheim JL Hankinson SE Helzlsouer KJ Holford TR Huang HY Moysich KB Tessari JD Wolff MS Zheng T Hunter DJ 1,1-Dichloro-2,2- bis(p-chlorophenyl)ethylene and polychlorinated biphenyls and breast cancer: combined analysis of five U.S. studies J Natl Cancer Inst 2001 93 768 776 10.1093/jnci/93.10.768 11353787

[B84] Weiderpass E Pukkala E Kauppinen T Mutanen P Paakkulainen H Vasama-Neuvonen K Boffetta P Partanen T Breast cancer and occupational exposures in women in Finland Am J Ind Med 1999 36 48 53 10.1002/(SICI)1097-0274(199907)36:1<48::AID-AJIM7>3.0.CO;2-2 10361586

[B85] Furberg H Millikan RC Geradts J Gammon MD Dressler LG Ambrosone CB Newman B Environmental factors in relation to breast cancer characterized by p53 protein expression Cancer Epidemiol Biomarkers Prev 2002 11 829 835 12223426

[B86] Zheng T Holford TR Mayne ST Luo J Owens PH Zhang B Zhang W Zhang Y Radiation exposure from diagnostic and therapeutic treatments and risk of breast cancer Eur J Cancer Prev 2002 11 229 235 10.1097/00008469-200206000-00006 12131656

[B87] Freedman DM Dosemeci M McGlynn K Sunlight and mortality from breast, ovarian, colon, prostate, and non-melanoma skin cancer: a composite death certificate based case–control study Occup Environ Med 2002 59 257 262 10.1136/oem.59.4.257 11934953 PMC1740270

[B88] Sanchez-Zamorano LM Flores-Luna L Angeles-Llerenas A Romieu I Lazcano-Ponce E Miranda-Hernandez H Mainero-Ratchelous F Torres-Mejía G Healthy lifestyle on the risk of breast cancer Cancer Epidemiol Biomarkers Prev 2011 20 5 912 922 10.1158/1055-9965.EPI-10-1036 21335508

[B89] Smith-Warner SA Spiegelman D Yaun SS Van Den Brandt PA Folsom AR Goldbohm RA Graham S Holmberg L Howe GR Marshall JR Miller AB Potter JD Speizer FE Willett WC Wolk A Hunter DJ Alcohol and breast cancer in women: a pooled analysis of cohort studies JAMA 1998 279 535 540 10.1001/jama.279.7.535 9480365

[B90] Chlebowski RT Blackburn GL Thomson CA Nixon DW Shapiro A Hoy MK Goodman MT Giuliano AE Karanja N McAndrew P Hudis C Butler J Merkel D Kristal A Caan B Michaelson R Vinciguerra V Del Prete S Winkler M Hall R Simon M Winters BL Elashoff RM Dietary fat reduction and breast cancer outcome: interim effi cacy results from the Women’s Intervention Nutrition Study J Natl Cancer Inst 2006 98 24 1767 1776 10.1093/jnci/djj494 17179478

[B91] Lee I-M Oguma Y Schottenfeld D, Fraumeni JF Physical activity Cancer Epidemiology and Prevention 2006 3 New York, NY: Oxford University Press 449 467

[B92] Smith BL Gadd MA Lawler C MacDonald DJ Grudberg SC Chi FS Carlson K Comegno A Souba WW Perception of breast cancer risk among women in breast center and primary care settings: correlation with age and family history of breast cancer Surgery 1996 120 2 297 303 10.1016/S0039-6060(96)80301-1 8751596

[B93] Grande GE Hyland F Walter FM Kinmonth AL Women’s views of consultations about familial risk of breast cancer in primary care Patient Educ Couns 2002 48 3 275 282 10.1016/S0738-3991(02)00035-6 12477612

[B94] Haas JS Kaplan CP Des Jarlais G Gildengoin V Pérez-Stable EJ Kerlikowske K Perceived risk of breast cancer among women at average and increased risk J Womens Health 2005 14 9 845 851 10.1089/jwh.2005.14.845 16313212

[B95] Emery JD Walter FM Ravine D Family history: the neglected risk factor in disease prevention Med J Aust 2010 192 12 677 678 20565342 10.5694/j.1326-5377.2010.tb03699.x

[B96] Walter FM Emery JD Genetic advances in medicine: has the promise been fulfilled in general practice? Br J Gen Pract 2012 62 596 120 121 10.3399/bjgp12X629955 22429411 PMC3289799

[B97] Murff HJ Byrne D Haas JS Puopolo AL Brennan TA Race and family history assessment for breast cancer J Gen Intern Med 2005 20 1 75 80 10.1111/j.1525-1497.2004.40112.x 15693932 PMC1490028

[B98] Greenhalgh T Robert G Macfarlane F Bate P Kyriakidou O Diffusion of innovations in service organizations: systematic review and recommendations Milbank Q 2004 82 4 581 629 10.1111/j.0887-378X.2004.00325.x 15595944 PMC2690184

[B99] Gilbert R Woodman J Allister J Rafi I De Lusignan S Belsey J Petersen I A simple approach to improve recording of concerns about child maltreatment in primary care records: developing a quality improvement intervention Br J Gen Pract 2012 62 600 478 486 10.3399/bjgp12X652346 PMC3381274 22781996

[B100] Bankhead C Emery J Qureshi N Campbell H Austoker J Watson E New developments in genetics-knowledge, attitudes and information needs of practice nurses Fam Pract 2001 18 5 475 486 10.1093/fampra/18.5.475 11604367

[B101] Tai TW Anandarajah S Dhoul N de Lusignan S Variation in clinical coding lists in UK general practice: a barrier to consistent data entry? Inform Prim Care 2007 15 3 143 150 18005561 10.14236/jhi.v15i3.652

[B102] de Lusignan S Mimnagh C Breaking the first law of informatics: the Quality and Outcomes Framework (QOF) in the dock Inform Prim Care 2006 14 3 153 156 17288700 10.14236/jhi.v14i3.625

[B103] Woodman J Allister J Rafi I de Lusignan S Belsey J Petersen I Gilbert R RCGP Multisite Safeguarding Audit A simple approach to improve recording of concerns about child maltreatment in primary care records: developing a quality improvement intervention 2012 62 600 e478 e486 10.3399/bjgp12X652346 PMC3381274 22781996

[B104] Wilson BJ Torrance N Mollison J Wordsworth S Gray JR Haites NE Grant A Campbell MK Miedyzbrodzka Z Clarke A Watson MS Douglas A Improving the referral process for familial breast cancer genetic counselling: findings of three randomised controlled trials of two interventions Health Technol Assess 2005 9 3 1 126 15694064 10.3310/hta9030

[B105] Emery J Morris H Goodchild R Fanshawe T Prevost AT Bobrow M Kinmonth AL The GRAIDS Trial: a cluster randomised controlled trial of computer decision support for the management of familial cancer risk in primary care Br J Cancer 2007 97 4 486 493 10.1038/sj.bjc.6603897 17700548 PMC2360348

[B106] Rubinstein WS Acheson LS O’Neill SM Ruffin MT 4th Wang C Beaumont JL Rothrock N Family Healthware Impact Trial (FHITr) Group Clinical utility of family history for cancer screening and referral in primary care: a report from the Family Healthware Impact Trial Genet Med 2011 13 11 956 965 10.1097/GIM.0b013e3182241d88 22075527 PMC3425444

