# Supplementary material for: Improving the management of people with a family history of breast cancer in primary care: before and after study of audit-based education
Source: BMC Fam Pract. 2013 Jul 24;14:105. doi: 10.1186/1471-2296-14-105 (PMC3734209; doi:10.1186/1471-2296-14-105)
Supplement: Additional file 1 — Box S1. Epidemiology of breast cancer in the UK. Box S2. Audit-based education (ABE) – a quality improvement method. The quality improvement strategy developed by the Primary Care Data Quality Programme (PCDQ). Table S1. Age-sex profiles of participating practices. Table S2. Growth in practice populations between first and second data collections. Table S3. Availability of key data fields in first and second data collections. [file 1471-2296-14-105-S1.docx]

**Supplementary data file**

**Box S1: Epidemiology of breast cancer in the UK**

**Box S2: Audit-based education (ABE) – a quality improvement method**

The quality improvement strategy developed by the Primary Care Data Quality Programme (PCDQ)

**Table S1: Age-sex profiles of participating practices**

**Table S2: Growth in practice populations between first and second data collections**

**Table S3: Availability of key data fields in first and second data collections**

**Box S1: Epidemiology of breast cancer in the UK**

- Despite being rare in men, breast cancer is now the most common cancer in the UK having more incident cases than lung or colorectal cases. It accounts for 31% of all new cases of cancer in females. 80% of breast cancers are diagnosed in women aged 50 and over.
- The lifetime risk of developing breast cancer is estimated to be 1 in8 for women and 1 in 1,014 for men in the UK.
- Although very few cases of breast cancer occur in women in their teens or early 20s, breast cancer is the most commonly diagnosed cancer in women under 35.
- Breast cancer accounts for around 16% of female deaths from cancer in the UK and was the most common cause of death from cancer in women until 1998.
- Breast cancer survival rates are better the earlier the cancer is diagnosed.
- Approximately 90% women diagnosed with stage I breast cancer survive beyond five years. This drops to around 10% if diagnosed with stage IV.
- The strongest risk factor for breast cancer (after gender) is age.

**Box S2: Audit-based education (ABE) – a quality improvement method**

The quality improvement strategy developed by the Primary Care Data Quality Programme (PCDQ)

1. **Identifying relevant best-evidence.** We identify guidance or other source of best-evidence for the planned intervention. We develop an ontology of the key concepts and how they might be represented in clinical records.
2. **Anonymised computer data.** Anonymised extraction from computerised electronic patient record systems (EPR) of the dataset required to report whether there is a gap between quality and best-evidence at baseline; and subsequently to report any quality improvement. The usual components are
   1. Denominator to allow standardisation of prevalence.
   2. Subset of people with the target condition – to create a virtual disease register.
   3. Clinically relevant co-morbidities, risk factors and treatment.
3. **Creating educational material to feedback at a Data Quality Workshop (DQW).** Processing that data to make it informative and providing comparative feedback combined with academic detailing. A key feature is presenting comparative feedback comparing practices at twice yearly meetings held within a locality / primary care organisation. These meetings are called Data Quality Workshops (DQW), generally locally led with educational outreach provided by a consultant/specialist of the relevant discipline also attending as a specialist/expert resource.
4. **Additional academic detailing.** In addition to the presentation at the DQW, practices are provided academic detailing in the form of two additional printed aids:
   1. “Laminate” – a single laminated A4 page summary of the practice demographics and case ascertainment compared with others who attended the DQW. This is for the practice notice board or another prominent location (we recommend wherever they take their breaks).
   2. Workbook – a slide by slide explanation of the DQW presentation – and what the data means for their practice, compared with their peers and any evidence-based guidance.
5. **List of patients requiring intervention (within participant practices only).** Running local searches in the practices to provide lists of patients that need to be targeted for intervention. These lists are usually generated for each individual GP. Experiential learning is that audit lists of up to 150 per 10,000 registered patients result in change; around 20 to 30 cases per GP.
6. **Supporting educational material and guidelines.** Providing/ providing access to and/or links to relevant evidence-based guidance and/or reviews
7. **Improved data recording.** Supporting the development of data entry forms and providing clinical coding or other training as required to overcome any barriers to EPR use or date recording.
8. **Feedback.** Participants are encouraged to give feedback and contribute to the future development of the ABE programme.

ABE is an intervention developed over 10 years ago; its aim is to provide feedback about performance against guidance. ABE includes feedback about quality compared with peers in a workshop setting usually led by a local GP with a specialist available as an expert resource, and also supported by academic detailing. ABE usually also identified lists of patients within the practices needing intervention,

**Table S1: Age-sex profiles of participating practices**

| **Age-** | **ALL** |  |  | **Practice 1** | |  | **Practice 2** | |  | **Practice 3** | |  | **Practice 4** | |  | **Practice 5** | |  | **Practice 6** | |  |
| --- | --- | --- | --- | --- | --- | --- | --- | --- | --- | --- | --- | --- | --- | --- | --- | --- | --- | --- | --- | --- | --- |
| **Band** | **Female** | **Male** | **Total** | Female | Male | **Total** | Female | Male | **Total** | Female | Male | **Total** | Female | Male | **Total** | Female | Male | **Total** | Female | Male | **Total** |
| 0-4 | **1155** | **1186** | **2341** | 135 | 166 | **301** | 288 | 272 | **560** | 160 | 199 | **359** | 100 | 99 | **199** | 228 | 221 | **449** | 244 | 229 | **473** |
| 5-9 | **931** | **1012** | **1943** | 98 | 150 | **248** | 206 | 175 | **381** | 167 | 176 | **343** | 89 | 105 | **194** | 207 | 213 | **420** | 164 | 193 | **357** |
| 10-14 | **846** | **916** | **1762** | 84 | 91 | **175** | 151 | 161 | **312** | 167 | 193 | **360** | 90 | 100 | **190** | 185 | 190 | **375** | 169 | 181 | **350** |
| 15-19 | **848** | **960** | **1808** | 90 | 113 | **203** | 134 | 139 | **273** | 194 | 201 | **395** | 110 | 132 | **242** | 160 | 183 | **343** | 160 | 192 | **352** |
| 20-24 | **1474** | **1128** | **2602** | 349 | 143 | **492** | 367 | 272 | **639** | 187 | 190 | **377** | 108 | 108 | **216** | 181 | 194 | **375** | 282 | 221 | **503** |
| 25-29 | **2332** | **1709** | **4041** | 521 | 285 | **806** | 875 | 638 | **1513** | 162 | 158 | **320** | 96 | 82 | **178** | 195 | 226 | **421** | 483 | 320 | **803** |
| 30-34 | **2098** | **1771** | **3869** | 391 | 268 | **659** | 800 | 725 | **1525** | 144 | 142 | **286** | 84 | 83 | **167** | 227 | 241 | **468** | 452 | 312 | **764** |
| 35-39 | **1402** | **1524** | **2926** | 252 | 217 | **469** | 416 | 493 | **909** | 146 | 160 | **306** | 83 | 92 | **175** | 182 | 242 | **424** | 323 | 320 | **643** |
| 40-44 | **1189** | **1287** | **2476** | 197 | 163 | **360** | 262 | 335 | **597** | 157 | 173 | **330** | 104 | 90 | **194** | 219 | 245 | **464** | 250 | 281 | **531** |
| 45-49 | **1013** | **1049** | **2062** | 159 | 121 | **280** | 191 | 209 | **400** | 168 | 172 | **340** | 101 | 116 | **217** | 185 | 182 | **367** | 209 | 249 | **458** |
| 50-54 | **774** | **821** | **1595** | 120 | 104 | **224** | 138 | 160 | **298** | 132 | 152 | **284** | 79 | 78 | **157** | 128 | 157 | **285** | 177 | 170 | **347** |
| 55-59 | **546** | **582** | **1128** | 106 | 90 | **196** | 79 | 91 | **170** | 103 | 108 | **211** | 49 | 69 | **118** | 67 | 85 | **152** | 142 | 139 | **281** |
| 60-64 | **482** | **457** | **939** | 101 | 90 | **191** | 70 | 72 | **142** | 87 | 78 | **165** | 33 | 38 | **71** | 77 | 58 | **135** | 114 | 121 | **235** |
| 65-69 | **359** | **304** | **663** | 79 | 45 | **124** | 37 | 37 | **74** | 59 | 59 | **118** | 40 | 34 | **74** | 55 | 47 | **102** | 89 | 82 | **171** |
| 70-74 | **335** | **281** | **616** | 66 | 43 | **109** | 44 | 43 | **87** | 60 | 47 | **107** | 38 | 29 | **67** | 33 | 34 | **67** | 94 | 85 | **179** |
| 75-79 | **259** | **192** | **451** | 39 | 36 | **75** | 27 | 24 | **51** | 62 | 51 | **113** | 24 | 12 | **36** | 43 | 19 | **62** | 64 | 50 | **114** |
| 80-84 | **195** | **151** | **346** | 23 | 19 | **42** | 20 | 13 | **33** | 56 | 40 | **96** | 21 | 22 | **43** | 20 | 16 | **36** | 55 | 41 | **96** |
| 85-89 | **104** | **51** | **155** | 19 | 7 | **26** | 11 | 7 | **18** | 34 | 17 | **51** | 6 | 3 | **9** | 12 | 3 | **15** | 22 | 14 | **36** |
| 90+' | **55** | **16** | **71** | 18 | 0 | **18** | 9 | 3 | **12** | 11 | 6 | **17** | 0 | 2 | **2** | 3 | 1 | **4** | 14 | 4 | **18** |
|  | **16397** | **15397** | **31794** | **2847** | **2151** | **4998** | **4125** | **3869** | **7994** | **2256** | **2322** | **4578** | **1255** | **1294** | **2549** | **2407** | **2557** | **4964** | **3507** | **3204** | **6711** |

**Table S2: Growth in practice populations between first and second data collections (Includes Under 18 years)**

|  | **Mean age** | **Standard deviation (SD)** | **% Female** | **% Male** | **Practice population (n)** |
| --- | --- | --- | --- | --- | --- |
| **First data collection** |  |  |  |  |  |
| Practice 1 | 35.26 | 19.21 | 55.7 | 44.3 | 4360 |
| Practice 2 | 32.22 | 16.18 | 51.8 | 48.2 | 7355 |
| Practice 3 | 35.85 | 22.45 | 49.3 | 50.7 | 4428 |
| Practice 4 | 33.60 | 21.03 | 48.5 | 51.5 | 2536 |
| Practice 5 | 32.15 | 19.82 | 49.1 | 50.9 | 4807 |
| Practice 6 | 34.59 | 18.95 | 54.3 | 45.7 | 3662 |
| **All** | **33.74** | **19.34** | **51.6** | **48.4** | **27148** |
| **Second data collection** |  |  |  |  |  |
| Practice 1 | 34.25 | 19.02 | 57.0 | 43.0 | 4998 |
| Practice 2 | 30.96 | 16.05 | 51.6 | 48.4 | 7994 |
| Practice 3 | 34.17 | 22.47 | 49.3 | 50.7 | 4578 |
| Practice 4 | 32.98 | 21.16 | 49.2 | 50.8 | 2549 |
| Practice 5 | 31.02 | 19.68 | 48.5 | 51.5 | 4964 |
| Practice 6 | 34.68 | 20.17 | 52.3 | 47.7 | 6711 |
| **All** | **32.90** | **19.47** | **51.6** | **48.4** | **31794** |

**Table S3 : Availability of key data fields in first and second data collections:**

|  | **First data**  **collection** | **Second data collection** |
| --- | --- | --- |
| Age | √ | √ |
| Gender | √ | √ |
| Ethnicity record | √ | √ |
| BMI (poor populated and is not included in analysis) | Query error, incomplete | √ |
| Smoking status records | √ | √ |
| Alcohol consumption records | √ | √ |
| Family history (all)** | √ | √ |
| Family history of cancer | √ | √ |
| Oestrogen | √ | √ |
| Oral contraception | √ | √ |
| Life style health education/advice** | √ | √ |
| Breast Cancer health education/advice | √ | √ |
| Term pregnancy** | √ | √ |
| Breast feed | √ | √ |

**Inconsistencies between round 1 and round 2 data collections
